# Spatio-Temporal Regularization for Longitudinal Registration to Subject-Specific 3d Template

**DOI:** 10.1371/journal.pone.0133352

**Published:** 2015-08-24

**Authors:** Nicolas Guizard, Vladimir S. Fonov, Daniel García-Lorenzo, Kunio Nakamura, Bérengère Aubert-Broche, D. Louis Collins

**Affiliations:** 1 Montreal Neurological Institute, McGill University, Montréal, Canada; 2 CENIR—ICM, Pitié Salpétrière, Paris, France; Universidad Carlos III de Madrid; Instituto de Investigación Sanitaria Gregorio Marañon, SPAIN

## Abstract

Neurodegenerative diseases such as Alzheimer's disease present subtle anatomical brain changes before the appearance of clinical symptoms. Manual structure segmentation is long and tedious and although automatic methods exist, they are often performed in a cross-sectional manner where each time-point is analyzed independently. With such analysis methods, bias, error and longitudinal noise may be introduced. Noise due to MR scanners and other physiological effects may also introduce variability in the measurement. We propose to use 4D non-linear registration with spatio-temporal regularization to correct for potential longitudinal inconsistencies in the context of structure segmentation. The major contribution of this article is the use of *individual template creation* *with* *spatio-temporal regularization of the deformation fields* for each subject. We validate our method with different sets of real MRI data, compare it to available longitudinal methods such as FreeSurfer, SPM12, QUARC, TBM, and KNBSI, and demonstrate that *spatially local temporal regularization* yields more consistent rates of change of *global structures* resulting in better statistical power to detect significant changes over time and between populations.

## Introduction

Longitudinal measures of brain volumetry are powerful tools to assess the anatomical changes underlying on-going neurodegenerative processes. In different neurological disorders, such as multiple sclerosis (MS), Alzheimer’s disease (AD) and Parkinson’s disease (PD), brain atrophy has been shown to be a good surrogate marker of disease progression[1–3]. Magnetic resonance imaging (MRI) can provide reproducible 3D structural images of the brain, which can be used to assess its integrity. Furthermore, the emergence of freely available longitudinal MRI databases, (e.g.,Alzheimer’s Disease Neuroimaging Initiative (ADNI)[4], Open Access Series of Imaging Studies(OASIS)[5] and others) provide the necessary data to develop and test new methods and investigate the longitudinal structural changes of healthy and pathological brains.

**Fig 1:**
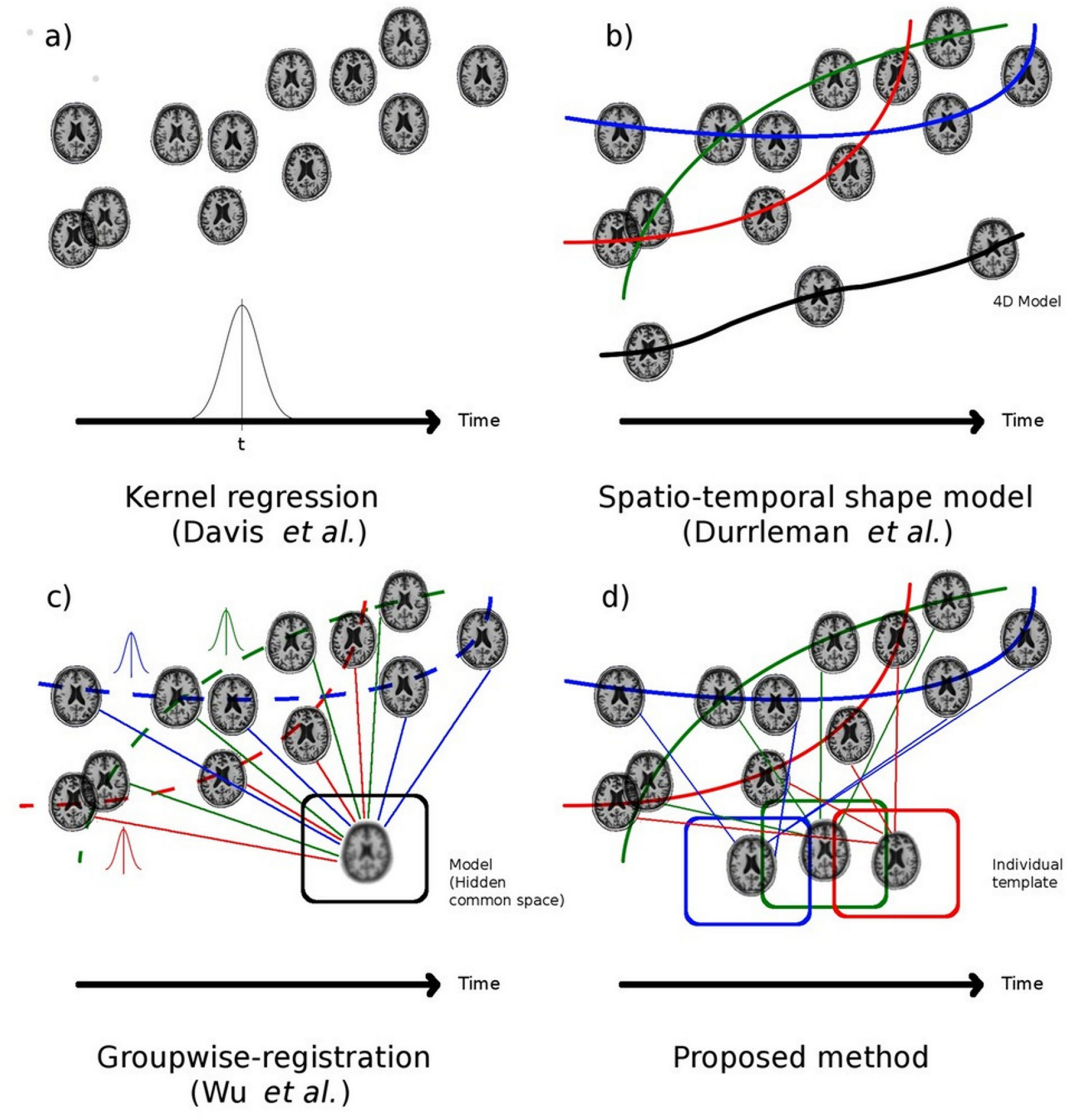
Longitudinal registration and template creation methods. Each vignette (a, b, c and d) represents different strategies proposed to overcome longitudinal MRI data analysis. The x-axis represents the time and the y-axis represents the anatomical variability of the image. Each subject’s time-points are connected by a colored line (blue, green and red) and the black line represents a longitudinal (4D) model. The square boxes represent the population 3D template in black and individual 3D templates in blue, green and red.

Image processing in MRI-based neuro-anatomical studies is often performed in a cross-sectional manner where each time-point is evaluated independently. Typically, brain morphometry comparisons can be done by matching paired images (template-to-subject or subject-to-subject), where the deformation field is used to map atlas regions or to compute voxel-wise comparisons of anatomical changes as in deformation-based morphometry (DBM). However, in the context of longitudinal datasets, the robust estimation of anatomical changes is still challenging [6]. Indeed, in the case of neurodegeneration occurring in a short period of time (2–3 years), if we assume that longitudinal changes are smoothly varying, spatially local, and temporally monotonic processes, considering individual time-points independently can generate unnecessarily noisy longitudinal measurements due to the intrinsic noise associated with each visit. Different studies have shown the impact of the MRI acquisition protocol on structural measurements [7] and cortical thickness [8]. Therefore, methods that integrate constraints from the temporal dimension (i.e., 4D methods) should produce more accurate, robust and stable measures of the longitudinal anatomical changes resulting in a more realistic estimation of temporal evolution. Different approaches have been proposed to overcome the complexity of anatomical 4D longitudinal data image analysis. We classify these methods in 2 major groups: “4D” and “longitudinal 3D”. The 4D approaches treat the individual and/or group-wise longitudinal data as an ensemble and provide longitudinal models or measurements. They are mathematically sophisticated approaches that have been proposed in the context of modeling larger anatomical changes over time (i.e., growth over the span of childhood). For example, a 4D population model creation using Gaussian kernel regression has been suggested by Davis et al. [9] where each image is registered independently to a moving average, avoiding the creation of an explicit parameterized model of the longitudinal changes (Fig 1A). Kernel regression has also been used in the framework of the Large Deformation Diffeomorphic Metric Mapping (LDDMM) for brain shapes [10] (Fig 1B) and images [10–12]. Regarding intra-subject 4D registration, Lorenzi et al. [13] have proposed 4D non-linear registration via a global 4D deformation optimization scheme in the Demons registration framework. Finally, Wu et al. [14] introduced an implicit mean-shape of the population which could be used for individuals. Their approach maximizes the spatio-temporal correspondence and continuity from a set of temporal fibre bundles (Fig 1C).

The longitudinal 3D approaches include the adaptation of popular 3D/cross-sectional methodswith some longitudinal constraints or longitudinal pre-processing. For instance, in the context of clinical evaluation over a few years where anatomical changes are small and continuous, the use of 3D individual template targets have been proposed to perform non-linear registration [15–17] or tensor-based analyses (TBM) [18]. Indeed, to compare anatomical differences, 3D population templates have proven their importance for different applications such as mapping function, structure, or vasculature [19] and group comparisons [20]. While different techniques exist to create unbiased population templates for multi-subject cross-sectional studies [21, 22], few of these techniques have been developed for the creation of an individual 3D subject template. More recently, Reuter et al. [16] created a subject-specific 3D template for longitudinal analysis by computing the median image of the linearly registered images of the same subject from different time-points and this method is implemented within the longitudinal version of FreeSurfer (http://surfer.nmr.mgh.harvard.edu) [16]. In the continuity of their work on voxel-based morphometry (VBM) [23–25], Ashburner et al. [17] presented an unbiased “group-wise intra-subject” template with an iterative longitudinal non-uniformity correction, linear and non-linear diffeormorphic registration that is implemented in the Statistical Parametric Mapping 12 (SPM12) (http://www.fil.ion.ucl.ac.uk/spm). Aubert-Broche et al. [26] also proposed to use robust non-linear individual templates to perform tissue classification and segmentation of pediatric images.

Inspired by previous work and the need for longitudinal analysis, we propose to include spatio-temporal constraints to analyze longitudinal MRI volumes, combining the advantages of both 4D longitudinal and 3D longitudinal approaches. An iterative algorithm is presented to create subject-specific templates for structural segmentation (Fig 1D). The decomposition of the longitudinal deformation fields, similar to a Taylor series, enables local spatial constraints as well as temporal regularization. While the spatial constraints aim to preserve the anatomical consistency in the image, the voxel-wise temporal regularization tackles the potential longitudinal alteration of the images. The temporal regularization is achieved with a local voxel-wise linear regression of the deformation components over time, resulting in a more consistent global longitudinal deformation. In this article, we first evaluate the stability and robustness of our method with a scan-rescan dataset, then, we assess its power to analyze a longitudinal cohort from the ADNI database. We show that a weak local spatial constraint over time can have significant positive global effects to significantly reduce inter-visit variability in the measurement of structure volumes such as the lateral ventricles, hippocampi and brain parenchyma.

## Methods

The objective of the template creation algorithm is to find the non-linear transformations that minimize the anatomical shape differences between images to create the most representative average of the subject's anatomy. Processing is achieved in two steps. First, all data is processed cross-sectionally to bring each volume into stereotaxic space. Second, this data is used to build a subject-specific individual template. The method and notation is inspired from Fonov et al. [22] and Aubert-Broche et al. [26], and described in the following sections. The nomenclature is summarized in Table 1.

**Table 1:** Notation.

Symbol	Definition
*v*	Voxelposition *v* varying from 0 to *N*
*k*	Iteration *k*
*I*_*t*_ *(v)*	Subject’s set of images from different time-points *t*
Φ_*L*_(*v*)	Subject-specific linear template at voxel *v*
$\Phi_{NL}^{k}(v)$	Subject-specific non-linear template at voxel *v*and iteration *k*
$\psi_{t,\Phi }^{k}(v)$	Deformation field of time-point *t* to template Фat voxel *v*
$\varphi_{{}_{t,\Phi }}^{k}\left(v\right)$	Bias free deformation field of time-point *t* to template Фat voxel *v*
Ω_*v*_	Neighborhood or patchsurrounding voxel *v*
*T (v*,*t)*	Trajectory of voxel *v* at time *t*
ℑ*T*(*v*,*t*)	Jacobian matrix of voxel *v* at time *t*
*β*_*t*_ *(v)*	Non-uniformity field at voxel *v*

The Montreal Neurological Institute research ethics committee gave approval for this study. Two neuroimaging datasets ("scan-rescan" and "ADNI") were used anonymously to evaluate the proposed algorithm and all subjects gave informed consent. Further information about ADNI can be obtained from www.adni-info.org and in the Acknowledgments section.

### Cross-sectional pre-processing

All MRI data are pre-processed to reduce the effects of artifacts and noise. The standard deviation of the MRI Rician noise is estimated automatically and image redundancy is used to reduce the noise using a non-local patch-based technique [27]. A non-parametric estimation of the slow varying non-uniformity field corrects the intensity inhomogeneity produced by scanner radio-frequency coil variations [28]. In addition, linear histogram matching is performed between each subject and a reference image to normalize the image intensities between subjects/scans to a range between 0.0 and 100.0. The reference image was created to represent the ageing population brain anatomy from the AD cohort using the unbiased template creation approaches proposed by Fonov et al. [22]. Finally, to correct for variation in head position, orientation and size, an initial 9 parameter linear registration (translation, rotation and scale) is computed to bring each subject into the ICBM152 template stereotaxic space [29].

### Longitudinal processing

The subject-specific template is based on the work of Guimond et al. [21, 30], Joshi et al. [31] and Fonov et al. [22] where a template is created in two steps, first using linear registration and second, using non-linear registration with a spatio-temporal regularization.

#### Linear individual template

In order to refine the alignment of individual images and estimate global whole brain scale factors between the consecutive visits, we perform a hierarchical iterative linear registration. Starting with the individual stereotaxic image average as the initial target, the linear individual template (Φ_*L*_(*v*)) is then defined as the intensity average of the B-spline (order 4) interpolated individual visit scans after affine registration. For each subject, a twelve parameter affine registration [29], based on an intensity cross-correlation similarity measure, is performed between the time-points’(*t* = [0..*n*]) and the subject-specific template volumes at 32, 16, 8 and 4mm hierarchical step sizes.

#### Non-linear minimum deformation individual template

A non-linear subject-specific template Φ_*NL*_(*v*) is estimated with an iterative approach, similar to the linear template, but using non-linear registration in order to estimate the local deformation between the visits and the individual template. To create Φ_*NL*_(*v*), a minimum deformation template (MDT) approach is used as described by Fonov et al. [22]. However, here the MDT estimation is modified to account for spatio-temporal regularity constraints (described in 2.2.3) and the implementation of the 4D constraints is done in the framework of a 3D non-parametric vector field estimator using the Automatic Non-linear Image Matching and Anatomical Labeling (ANIMAL) procedure [32].

For the MDT, ANIMAL estimates the non-linear deformation field required to align two image volumes in a hierarchical manner, where the algorithm maximizes the local cross-correlation of the blurred image intensity of the source image with the equivalently blurred image intensity of a target image. Starting from down-sampled images, the displacement vectors that best match the two images are stored at the nodes of a 3D grid, producing a dense deformation field. Then, the deformation field is upsampled and used to initiate the deformation at the next hierarchical iteration where the blurring kernel is reduced, and the deformation field is refined. Details of the ANIMAL algorithm are described in[32, 33].

To satisfy the intensity constraint condition (Eq 1) and the deformation constraint condition of (Eq 2), we use an iterative approach. At each iteration, ANIMAL is used to map the voxels *v* from the MRI of a subject at time-point t = [0..n], *I*_*t*_(*v*), to the current evolving estimate of the template $\Phi_{NL}^{k}\left(v\right)$ at iteration *k* through a deformation transformation $\psi_{t,\Phi }^{k}(v)$. This is followed by the removal of the bias (or mean deformation $\sum_{t=0}^{n}\psi_{t,\Phi }^{k}\left(v\right)$) to obtain $\phi_{t,\Phi }^{k}(v)$ (Eq 3) (thus enforcing the condition in Eq 2) and calculating a new estimate of the template $\Phi_{NL}^{k+1}\left(v\right)$ (Eq 4).

$$\Phi_{NL}^{k}(v)=\underset{\Phi }{\mathrm{argmin}}\sum_{t=0}^{n}\int_{volume}{\left(\Phi_{I}^{k}\left(v\right)-I_{t}\left(\psi_{{}_{t,\Phi }}^{k}\left(v\right)\right)\right)}^{2}dv$$ (1)

$$\Phi_{NL}^{k}(v)=\underset{\Phi }{\mathrm{argmin}}\sum_{t=0}^{n}\int_{volume}^{}{\left|\bar{\psi_{t,\Phi }^{k}(v)}\right|}^{2}dv$$ (2)

$$\varphi_{{}_{t,\Phi }}^{k}\left(v\right)=\psi_{t,\Phi }^{k}\left(v\right)\circ \bar{\sum_{t=0}^{n}\psi_{t,\Phi }^{k}\left(v\right)}$$ (3)

$$\Phi_{NL}^{k+1}(v)=\frac{1}{n}\sum_{t=0}^{n}I_{t}\left(\varphi_{{}_{t,\Phi }}^{k}\left(v\right)\right)$$ (4)

In these equations, the operation ∘ denotes concatenation of transformations, and $\bar{X}$ denotes inversion of a transformation *X*.

The algorithm is initialized with the individual linear template (Φ_*L*_(*v*)). At each iteration *k*, *ψ*_*t*,Φ_ is the non-linear transformation required to map *I*_*t*_ to $\Phi_{NL}^{k}$ which was obtained using ANIMAL. It is spatially constrained with a linear elastic body model while it minimizes the intensity difference of the paired images (i.e., between template and time-point images). The linear elastic body constraints are justified in such intra-subject registrations where very large deformations are not expected. The parameters of the hierarchical non-linear registration are chosen to ensure that the transformation defined by the vector field is smooth, bijective and invertible [34]. The details of the iterative hierarchical scheduleand the non-linear registration parameters for the 3D grid step size, image blurring kernel and similarity measure neighborhood size are summarized in Table 2. The registration schedule parameters are similar to Fonov et al. [34] and ANIMAL is robust to changes in parameters by a factor of 2[32, 35].

This subject-specific template creation process yields the non-linear deformations to map each of the subject time-points toward the template. By concatenating a forward transformation to the template and the inverse transformation toward a specific time-point, we can obtain the total non-linear transformation between two time-points transitively.

**Table 2:** ANIMAL non-linear registration schedule. For each iteration, we define a step size as the distance between control nodes for the free-form deformation recovered. The blurring kernel is the size of the full-width-half-maximum of the Gaussian kernel used to blur the source and target data. The local correlation which defines the local similarity is estimated in the neighborhood of diameter equals to the neighborhood size parameter.

Iteration	Step size(mm)	Blurring kernel (mm)	Neighborhood size (mm)
1	16	8	48
2–3	8	4	24
4–5	4	2	12
6–7	2	1	6
8–9	1	1	6

#### Spatio-temporal regularization of minimum deformation template

The MDT algorithm described above is modified to include an additional constraint for the non-linear transformations between time-points. It is implemented as an additional regularization step which is performed at each iteration of the template creation in the spatio-temporal domain in order to obtain a smooth non-linear deformation over time, since we expect the anatomical changes to happen in a slow and continuous fashion. We replace the individual time-point non-linear registrations $\psi_{t,\Phi }^{k}$ with a continuous and smooth transformation field $T(v,t)=[\psi {\left(v\right)}_{t_{0},\phi }^{k},\ldots ,\psi {\left(v\right)}_{t_{n},\phi }^{k}]$ where *T(v*,*t)*can be seen as the trajectory of voxel position *v* over time *t*.The proposed spatio-temporal regularization of the longitudinal deformation field is achieved through the following steps:

First, we decompose the longitudinal deformation component of the transformation into a simplified Taylor series expansion of order 1 in space, where the higher order terms are neglected, which allows for spatial regularization, such as:

T(v+Δv,t)≈T(v,t)+ℑT(v,t)⋅Δv (5)

This Taylor expansion presents the advantage of accounting for the longitudinal deformation (or temporal trajectory, *T*(*v*,*t*)) as well as the longitudinal local variation (Jacobian matrix, ℑ*T*(*v*,*t*)).

Second, we want to regularize the trajectory (*T*(*v*,*t*)) to obtain smooth longitudinal deformations while preserving the longitudinal local variation of the Jacobian matrix (ℑ*T*(*v*,*t*)), such as:

$$\begin{array}{l} ℑT\left(v,t\right)=\left[\begin{array}{ccc} \frac{\partial T{\left(v,t\right)}_{1}}{\partial v_{1}} & \ldots & \frac{\partial T{\left(v,t\right)}_{1}}{\partial v_{3}}\\ \vdots & & \vdots \\ \frac{\partial T{\left(v,t\right)}_{3}}{\partial v_{1}} & \cdots & \frac{\partial T{\left(v,t\right)}_{3}}{\partial v_{3}} \end{array}\right]\approx \\ \left[\begin{array}{ccc} \frac{T{\left(v+\Delta v,t\right)}_{1}-T{\left(v-\Delta v,t\right)}_{1}}{2\cdot \Delta v_{1}} & \ldots & \frac{T{\left(v+\Delta v,t\right)}_{1}-T{\left(v-\Delta v,t\right)}_{1}}{2\cdot \Delta v_{3}}\\ \vdots & & \vdots \\ \frac{T{\left(v+\Delta v,t\right)}_{3}-T{\left(v-\Delta v,t\right)}_{3}}{2\cdot \Delta v_{1}} & \cdots & \frac{T{\left(v+\Delta v,t\right)}_{3}-T{\left(v-\Delta v,t\right)}_{3}}{2\cdot \Delta v_{3}} \end{array}\right] \end{array}$$ (6)

To preserve the spatial consistency, we approximate the Jacobian matrix ℑ*T*(*v*,*t*) from Eq 6, by averaging across finite differences, such as:

$$T\left(v+\Delta v,t\right)\approx T\left(v,t\right)+\frac{1}{\left|\Omega_{v}\right|}\sum_{u\in \Omega_{v}}ℑT\left(u,t\right)\cdot \Delta v$$ (7)

where Ω_*v*_ is the local neighbourhoodcentered on *v*. Thus, this approximation provides a spatially regularized longitudinal deformation and in our experiments, we found that a 3x3x3 local neighbourhood was a good comprise between spatial smoothing and computational time.

Simultaneously, we perform linear regression of the zeroth order term in Eq 5 in the temporal domain such as:

$$T\left(v,t\right)\approx T_{0}(v)+T_{1}(v)\cdot t$$ (8)

where *T*_0_(*v*) is the intercept and *T*_1_(*v*) is the slope of the linear regression.

Thus, we effectively perform spatio-temporal regularization of the set of deformations fields with a spatial regularization (Eq 6) and a temporal regression (Eq 8), such as:

$$T^{*}\left(v,t\right)=T_{0}\left(v\right)+T_{1}\left(v\right)\cdot t+\frac{1}{\left|\Omega_{v}\right|}\sum_{u\in \Omega_{v}}ℑT\left(u,t\right)\cdot \Delta v$$ (9)

We use the resulting regularization procedure instead of Eq 3 in the MDT template creation.

This approach presents the advantage of taking into consideration the longitudinal deformation at each voxel and at the local neighbourhood level by the means of the local Jacobian matrix and the explicit local voxel-wise regularization of the deformation field components.

#### Individual template-based bias field correction

Intensity non-uniformity may vary between longitudinal scans due to differences in field inhomogeneity (*B*_*1*_) and receiver coil sensitivity [36]as well as differences in the positioning of the subject inside the coil. As described by Holland et al.[37] as well as Ashburner and Ridgway [17], if uncorrected, these temporal intensity non-uniformities could be detected as atrophy or growth with intensity-based non-linear registration tools. Therefore, inspired by the differential intensity inhomogeneity correction proposed by Lewis et al. [38], we propose to use the intensity difference of the subject-specific template and the warped time-point image to estimate the smooth longitudinal inhomogeneity correction field with *N*_3_[28]. *N*_3_ iteratively sharpens the histogram of the image intensity difference by de-convolving Gaussian fields from the true signal, while using splines to represent the estimated bias field. During the iterative process of the individual template creation and after the spatio-temporal regularization, the image intensity difference of the subject visit (*I*_*t*_) and the current template (Φ_*NL*_(*v*)) is computed at each iteration after resampling *I*_*t*_ with the transformation $\psi_{{}_{t,\Phi }}^{k}$. The bias field for each visit ($\beta_{t}^{k}$) is estimated from the differential image (Eqs 10 and 11).

$$\alpha_{t}^{k}=N_{3}\left(I_{t}^{k}\left(\psi_{{}_{t,\Phi }}^{k}\left(v_{i}\right)\right)-\Phi_{I}^{k}(v_{i})\right)$$ (10)

$$\beta_{t}^{k}=\alpha_{t}^{k}/\exp\left(\frac{1}{n}\sum_{t=0}^{n}\log\left(\alpha_{t}^{k}\right)\right)$$ (11)

Then the bias field is transformed back into the native time-point space to correct the residual longitudinal inhomogeneity of the source images for the following iteration (Eq 11).

$$I_{t}^{k+1}(v)=I_{t}^{k}\left(v\right)\cdot \beta_{t}^{k}\left(\bar{\psi^{k}{}_{t,\Phi }(v_{i})}\right)$$ (12)

#### Optimization and convergence

The non-linear template creation optimization is done at 5 hierarchical levels, starting with deformations estimated every 16, 8, 4, 2 and finally 1mm and the corresponding non-linear registration parameters are summarized in Table 2. At each level, the regularizations are performed consecutively in the order of Eqs 2, 9 and 3.An initial spatial regularization is applied to the subject visit-template deformation with a Gaussian kernel while for the spatio-temporal regularization, the whole time series deformation set is constrained (Eq 9). In our implementation, different parameters of the spatio-temporal regularization can be adjusted. The neighborhood size of the Jacobian matrix computation can be increased to obtain smoother deformations.

In previous cross-sectional template creation studies, we found that 9 iterations are enough for the convergence of the iterative process at each hierarchical level[22]. In the case of individual template creation, the additional longitudinal regularization could slow down convergence but it is compensated by the anatomical similarity of the images being registered.We found in our experiments that 9 iterations are thus also sufficient to converge. The template, longitudinal non-uniformity correction and deformation fields estimated at one hierarchical level are all used to initialize the procedure at the next hierarchical level.

### Experiments

#### Data

Two neuroimaging datasets were used anonymously to evaluate the proposed algorithm: *Scan-rescan* and *ADNI*.

First, to evaluate stability and potential bias, a scan-rescan database of 20 healthy subjects scanned 4 times within the same week (twice during a first session and twice again over 2 different days) was used. Each subject was taken out from the scanner before getting back in for each rescan session. No volume change is expected for the subjects in this database. The T1-weighted MRI images were acquired on a 1.5T SIEMENS MRI scanner with a 3D spoiled gradient echo (GRE) sequence (TR = 22ms, TE = 9.2ms, flip angle = 30°, 1mm isotropic voxels).

Second, to evaluate the performance of the algorithm when changes over time are expected, we used data obtained from the publically available ADNI database (adni.loni.usc.edu). The ADNI was launched in 2003 by the National Institute on Aging (NIA), the National Institute of Biomedical Imaging and Bioengineering (NIBIB), the Food and Drug Administration (FDA), private pharmaceutical companies and non-profit organizations, as a $60 million, 5-year public private partnership. The primary goal of ADNI has been to test whether serial MRI, positron emission tomography (PET), other biological markers, and clinical and neuropsychological assessment can be combined to measure the progression of mild cognitive impairment (MCI) and early AD. Determination of sensitive and specific markers of very early AD progression is intended to aid researchers and clinicians to develop new treatments and monitor their effectiveness, as well as lessen the time and cost of clinical trials. More information about the ADNI investigators is given in the Acknowledgment section.

To date these three protocols have recruited over 1500 adults, ages 55 to 90, to participate in the research, consisting of cognitively normal older individuals, people with early or late MCI, and people with early AD. The follow up duration of each group is specified in the protocols for ADNI-1, ADNI-2 and ADNI-GO. Subjects originally recruited for ADNI-1 and ADNI-GO had the option to be followed in ADNI-2. For up-to-date information, see www.adni-info.org.

From the website (www.adni.loni.ucla.edu/ADNI), ADNI-1 AD and normal controls (NC) subjects with 4 time-points (0, 6, 12 and 24 months) acquired on a 1.5T scanner that are part of the standardized set of subjects as described by Wyman et al.[39] were selected. This selection yielded155 NC (age average at baseline = 76.0±4.9 years) and 98 AD patients (age average at baseline = 75.3±7.3) that passed quality control[40]. The3D T1-MPRAGE images (TR = 2300–3000, TE = /3–4 ms, flip angle = 8–9°, section thickness = 1.2 mm, 256 reconstructed axial sections) with the following image pre-processing: gradient non-linearity distortion correction (grad-wrap [41]) and intensity non-uniformity (N3 [28]) were used for subsequent analysis.

#### Metrics

In order to evaluate the stability, regularity, continuity and bias of the proposed approach, we chose metrics based on ventricular, hippocampi and cerebral segmentations for each subject at each time-point. These structures were chosen since they have previously been used to represent the progression of neurodegenerative processes such as in MS or AD [38, 42, 43]. For the methods described below, these structures were either (i) segmented locally using the patch-based technique proposed initially by Coupé et al. [44] for hippocampus, for ventricles by Fonov et al. [8] and for brain Eskildsen et al. [45] combined with a Bayesian tissue classifier [46] to remove cerebrospinal fluid (CSF) from the initial brain mask to conserve only brain tissue; or (ii) data was downloaded from the "MRI image analyses" section of the ADNI website (www.loni.ucla.edu/ADNI) as indicated below.

#### Methods compared

The proposed method is compared to seven other methods. Like the proposed method, the first two are based on the ANIMAL non-linear registration framework, while the five others are based on publicly available methods that include FreeSurfer, SPM12, QUARC, TBM and KNBSI (http://sourceforge.net/projects/bsintegral). The eight techniques are identified as follows:

- Longitudinal individual template (LIT): LIT is the new method proposed in this paper, with spatio-temporal regularization with an individual template.
- Individual template (IT): IT method is like LIT with longitudinal pre-processing using all time-points, but without applying the spatio-temporal regularization.
- Cross-sectional (CS): CSmethod is based on ANIMAL, and uses direct linear registration [29] of each time-point independently to the common stereotaxic space (MNI template) after intensity non-uniformity correction.

**Fig 2:**
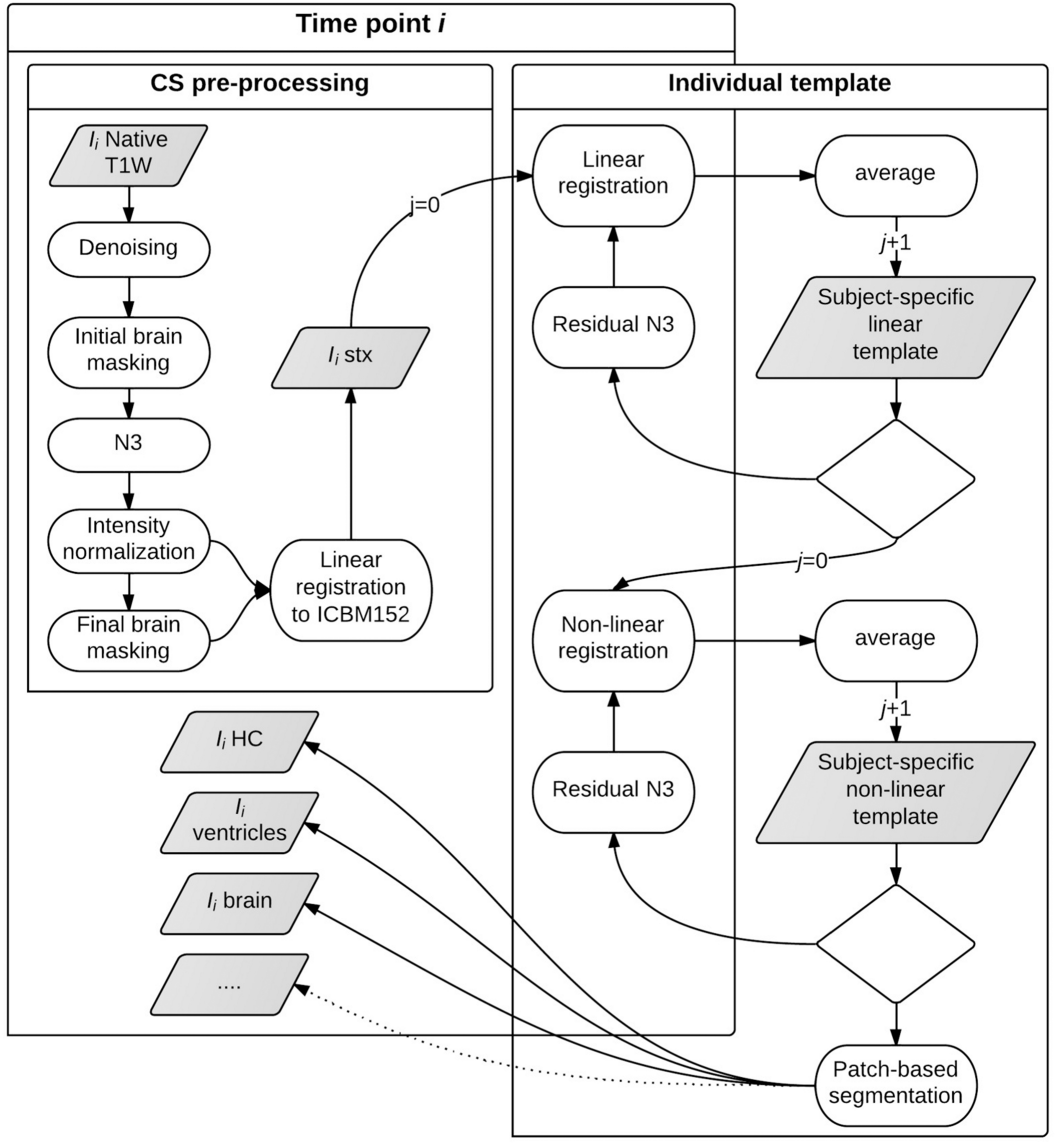
Longitudinal pipeline diagram. The different steps performed on each subject time-points are represented in the left part of the diagram, where the processes in the left small square represents the cross-sectional (CS) part of the pipeline. The individual template (IT) creation (linear and non-linear) is represented in the right side of the figure.

CS, IT and LIT represent different levels of the pipeline stages as seen in Fig 2 thus enabling an evaluation of the contribution of the longitudinal processing and the spatio-temporalregularizationsteps.

For CS, the structure segmentation was performed independently on the scan from each subject’s time-point and the volume change was estimated by computing the volume difference between the visits. In the case of the IT and LIT longitudinal approaches, only the subject-specific template was segmented and the estimated non-linear transformations were used to transform the segmentation to each time-point and estimate the Jacobian determinant. The volume change was estimated by integrating the Jacobian determinant within the regional structure masks for whole brain, ventricles and hippocampi.

- FreeSurfer (FS): The longitudinal stream of FSsoftware (version 5.1) was chosen as it has shown better results for longitudinal analyses than the cross-sectional version, except for longitudinal whole brain measurement [47]. FS provides structural segmentations of each subject time-point that are initialized by independent cross-sectional segmentations estimated from a linear individual template. For the scan-rescan analysis, the longitudinal version of FS was used to segment the hippocampi, lateral ventricles and brain. Briefly, FS processing included motion correction and averaging [48] of multiple volumetric T1 weighted images (when more than one was available), removal of non-brain tissue using a hybrid watershed/surface deformation procedure [49], automated Talairach transformation (Collins, 1994), intensity normalization [28] and segmentation of the subcortical white matter and deep gray matter volumetric structures (including hippocampi, amygdala, caudate, putamen, and ventricles) [50, 51]. For analysis of the ADNI data, we downloaded the appropriate values from the ADNI website (UCSF-Longitudinal FreeSurfer (5.1), 2014/05/01) as we felt that these would have been optimally run by the authors.
- Statistical Parametric Mapping 12 (SPM12): A unified model which combines intensity non-uniformity correction, linear registration and non-linear registration was proposed by Ashburner et al. [17] and implemented in SPM12. Their method produces a subject-specific template and uses the Jacobian determinants of the deformation map of the visit toward the template. As SPM12 does not create structure segmentations, our in-house segmentation tools were applied on the SPM12 subject-specific template and the volume change was estimated by integrating the Jacobian determinant within the regional structure masks for whole brain, ventricles and hippocampi. SPM12 was run locally for the scan-rescan and ADNI data.
- K-means clustering boundary shift integral (KNBSI): KNBSI [52] is based on the classic BSI procedure [53] and measures the tissue boundary displacement of a pair of images for the whole brain. KNBSI uses tissue specific normalization, k-means classifiers and specific parameters to account for large multi-site image intensity variability (in terms of SNR and tissue contrast differences). To account for the multiple tissue boundariesof the hippocampus, we used the double intensity windowing approach technique which estimates the boundary shift between CSF and grey matter as well as between grey and white matter [54].For the scan-rescan data, KNBSI was run locally for all structures after applying our in-house differential bias correction as recommended by the author. For the ADNI data, KNBSI data was downloaded from the ADNI site for whole brain and ventricles (Fox Lab, 2014-01-31), again to have optimally run values. We ran double window KNBSI locally for the hippocampi, as these values were not available on the ADNI website.
- Quantitative anatomical regional change (QUARC): QUARC [37] estimates the volume changes over a region defined in the baseline image where the deformation is estimated by combining pair-wise forward and backward non-linear transformations with intensity normalization. As QUARC is not publically available, we did not use it in the scan-rescan evaluation. However, for analysis of the ADNI data we downloaded QUARC results (UCSD, downloaded on 2014-06-01) from the ADNI web site.
- Tensor-based morphometry (TBM): TBM method proposed by Hua et al. [18], first estimates the statistical properties of the Jacobian determinant of non-linear deformations used to map training subjects to a population template. Second, a group of voxels with a significant rate of atrophy as measured by the Jacobian (p<0.001) in the temporal lobes are defined as a region of interest (“stat-ROI”). Finally, a single measurement for each subject, of an independent testing set, is obtained by integrating the Jacobian determinant of the non-linear deformations to the identical population template within the stat-ROI. TBM is not publically available and was not evaluated with the scan-rescan data. TBM results for ADNI data were downloaded from the ADNI website (USC, 2013-11-17).

Each image processing pipeline has a different level of robustness, and MRIs that do not pass quality control could adversely affect the estimation of statistical power. Instead of a head-to-head comparison, we decided to keep only datasets that passed visual quality control. For the data downloaded from the ADNI website, quality control information was only available for FS, KNBSI, and QUARC data. Subjects who passed quality control with the following arguments were kept for the power analyses: FS: QVERALLQC = “Pass” or “Partial”; for the ventricular KNBSI: BSI VENTACCEPT = 1, REGRATING ≤ 3, for KNBSI: KMNREGRATING ≤ 3; and QUARC QCPASS = 1. The final cohort number for each method is summarized in Table 3.

**Table 3:** Number of ADNI-1 subjects used for the power analyses for the different methods. These subjects were available from the downloaded results and/or passed quality controlfor each of their time-points (m0, m6, m12, m24).

Method	NC	AD
**CS**	153	95
**IT**	155	98
**LIT**	155	98
**FS**	152	96
**SPM12**	98	60
**KNBSI**	105	66
**QUARC**	131	73
**TBM**	115	73

#### Statistics

For the scan-rescan dataset, the percent volume change (*VC*) and the absolute percent volume change (*aVC*) were used respectively to evaluate bias and variability of structure volume (*V*). For each structure of each subject at time-point *t* of the n visitsand the structure average volume ($\frac{1}{n}\sum_{i=1}^{n}V_{i}$), *VC* and *aVC* were estimated with the following formulas:

$$VC_{t}=100*\left(1-\frac{V_{t}}{\frac{1}{n}\sum_{i=0}^{n}V_{i}}\right)\;\text{and}\;aVC_{t}=\left|VC_{t}\right|$$ (13)

The significant differences between the match-paired segmentations were compared with a paired t-test for the *VC* comparison and a Wilcoxon sign-rank test for the *aVC* comparison. The Wilcoxon signed rank test was chosen over a paired t-test because the scan-rescan *aVC* values do not follow a normal distribution due to the use of the absolute value.

For the longitudinal dataset, the percent volume change measures atrophy or growth using the baseline volume (*V*_0_) as a reference such as:

$$longVC_{t}=100*\left(1-\frac{V_{t}}{V_{0}}\right)$$ (14)

For the longitudinal results, we use power analyses to estimate the required sample size to assess the interaction of treatment and time in a longitudinal study where smaller longitudinal variability will enable better detection of a potential treatment effect. Here, the volume change was estimated using a linear mixed-effect model (LME). Indeed, linear mixed-effect modeling has shown to be a powerful statistical technique to analyze longitudinal data [55]. In this study, we used a simple LME of the volume changes (*longVC*) consisting of a temporal, time-point *t* interval (*Interval*_*It*_) and group (*Group*) fixed-effects while subject (*I*) was chosen as random effects, such as:

$$longVC_{It}^{}=(\beta_{1}+\beta_{2}\times Group+b_{I})\times Interval_{It}+\varepsilon_{It}$$ (15)

Power analyses, as described by Diggle et al. [56] and applied in Reuter et al. [16], for longitudinal analysis were performed to estimate the sample size. From the LME model estimation, the common variance (unexplained variability in *longVC*), the correlation of the repeated observations, the number of time-points, the smallest meaningful difference in the rate of change between AD and NC to be detected (effect size), the power of the test (here we chose 80%) and the within-subject variance of the time-points were used to compute sample size. Using the Diggle et al. [56] formula, power analysis was performed using the R software package (http://www.r-project.org) with the *lme4 and longpower* library. The 95% confidence intervals of the estimated sample sizes were obtained from 1000 parametric bootstrappings of the LME model.

The stability of LME model is influenced by the variability of the data as well as the number of time-points. Similarly, the power of the method is more influenced by the baseline and final time-point. Thus, only subjects with 4 time-points successfully passing the quality control were included for the power analysis (Table 3).

## Results

**Fig 3:**
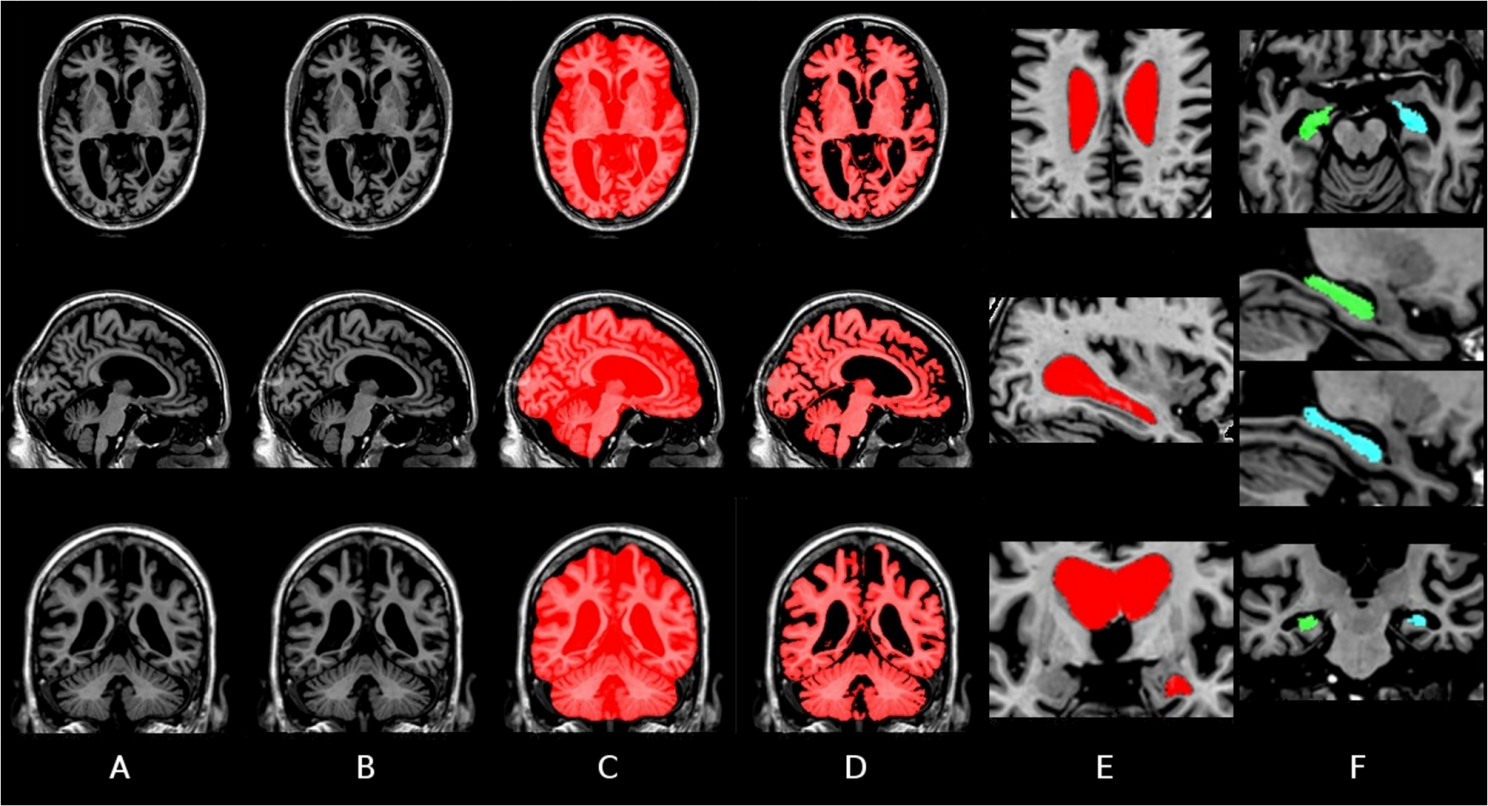
Individual LIT template segmentations of an AD subject from ADNI. Axial, sagittal and coronal slices are presented with from left to right: A) Linear individual template, B) non-linear individual template, C) BEaST skull-stripped mask, D) brain mask, E) lateral ventricle mask and F) right (blue) and left (green) hippocampus mask.

**Fig 4:**
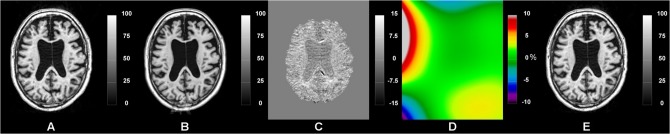
Individual longitudinal template-based bias field correction of an AD subject from the ADNI database. From left to right: A) baseline time-point, B) individual linear template, C) baseline time-point and individual template intensity difference image (or A-B), D) bias field of the difference image (C) and E) the baseline image after correction of the longitudinal bias field (D). (Note the different ranges on the color bars.)

Qualitatively, a general overview of the pipeline segmentation and individual template of one subject can be appreciated in Fig 3. Also, an example of individual template-based longitudinal non-uniformity intensity correction is depicted in Fig 4.

### Scan-rescan dataset

**Fig 5:**
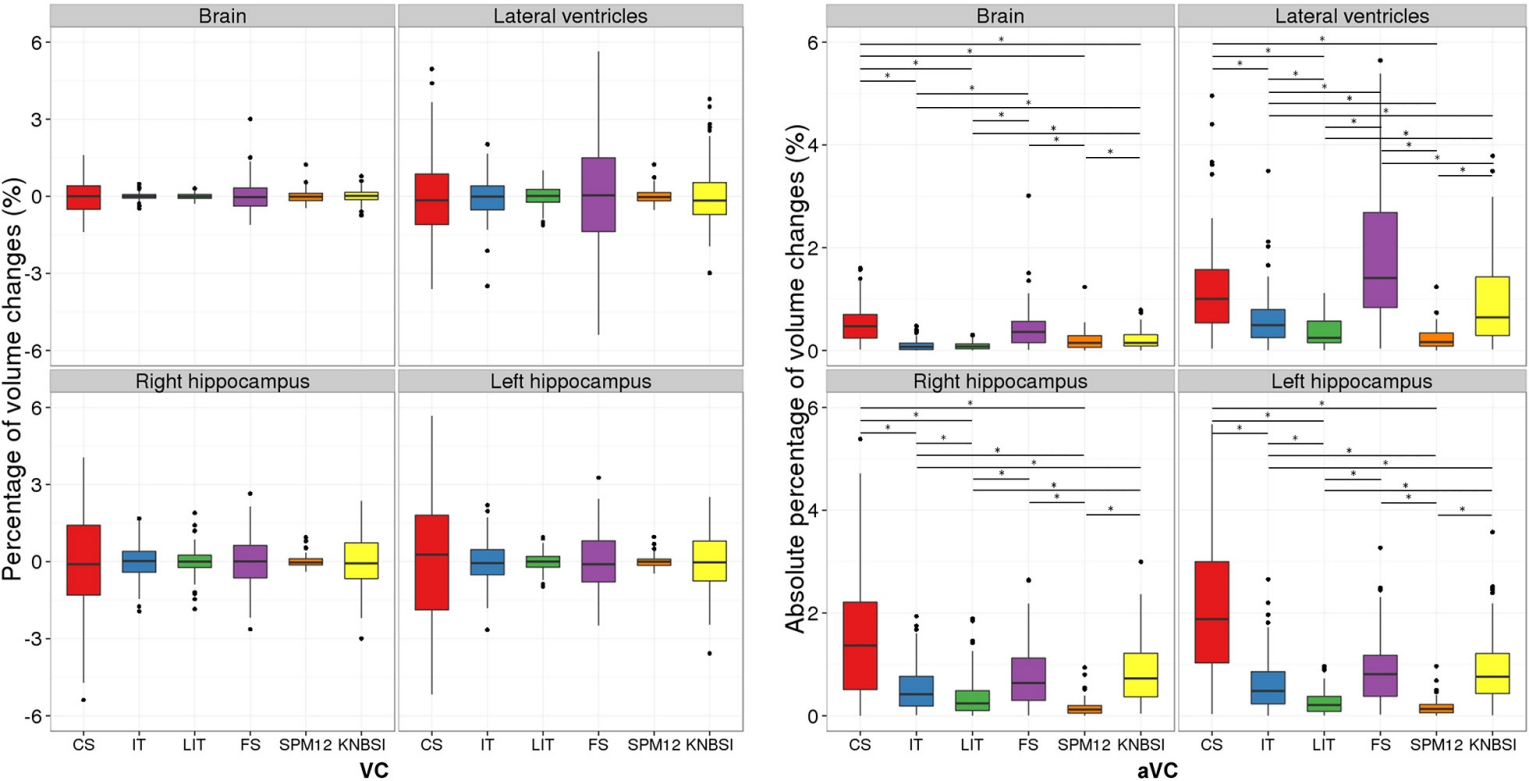
Brain, ventricle and left/right hippocampus percentage of volume change for the scan-rescan dataset for the different methods (CS, IT, LIT, FS, SPM12 and KNBSI). The significant difference (p<0.01) computed with a match paired Wilcoxon sign-rank are represented by a *where the pairs are represented by the thicker horizontal lines.

The scan-rescan dataset should show no anatomical variability since the 4 MRI scans were acquired during a week. Fig 5 shows the brain, ventricle and hippocampi volume changes (*VC* and *aVC*) for the cross-sectional (CS) and the longitudinal techniques (IT, LIT, FS, SPM12 and KNBSI) for the repeated sessions. For *VC* and *aVC*, the smallest structures present the highest volume variability. The method variability ranking is similar across structures excepted for FS and KNBSI, which show more variability for the lateral ventricles and the hippocampi measures.

No significant bias was found when comparing the mean *VC* of the different methods, as the mean *VC* values from all methods was centered on zero.

When looking at the *aVC*, the longitudinal methods (IT and LIT) significantly reduce (p<0.02) the variability for all the segmented structures compared to the cross-sectional (CS) method. The longitudinal pre-processing and registration methods such as SPM12 and LIT result in smaller variability between successive sessions compared to CS, IT, FS and KNBSI. Furthermore, SPM12 and LIT methods significantly reduce the *aVC* for the ventricle segmentation (p<0.02). The mean *aVC* respectively for the brain, ventricles and left/right hippocampi with the LIT approach are (in percent change): 0.093 (±0.073), 0.355 (±0.387), 0.279 (±0.277) and 0.416 (±0.432).

### Longitudinal dataset

The identical cohort of subjects from ADNI-1 was used to evaluate the different methods, however, subjects scans failing during pipeline processing or absent at the time of method result publication were removed from the analyses and the final number of subjects analysed is summarized in Table 3

Smaller longitudinal variability should improve the statistical power to detect changes in an individual, and facilitate detection of group differences (treatment effects) and thus reduce the number of subjects required for analysis in a clinical trial. In Table 4, we provide estimates of the different sample sizes required to detect a treatment effect that would reduce the annual AD atrophy rate by 25% for the different structures and methods. Table 4 also shows the annual atrophy rate for the different structures and methods. In general, amongst all methods compared, the LIT method requires a smaller number of subjects per arm for all treatment effect sizes for the brain and hippocampi measurements while KNBSI hold the smallest sample size for the lateral ventricles.

**Table 4:** Sample size per arm needed to detect a 25% reduction in the annualized rate of brain, ventricular and hippocampus volume change at 80% power for the different methods, while taking into account the normal rate of atrophy. The smallest detectable difference in the rate of change between AD and NC (effect size) and the estimated annual atrophy rate for the different structures for normal controls (NC) and Alzheimer subjects (AD) are also provided with the range representing the 95% confidence interval obtained from parametric bootstrapping of 1000 times.

						Annual atrophy rate in % [95% CI]			
Structure	Method	Sample size per arm[95% CI]		Effect size in %/year[95% CI]		NC		AD	
**Brain**	**CS**	>1000	[––]	-0.11	[-0.21–0.07]	-0.78	[-1.14–0.43]	-1.24	[-1.71–0.98]
	**IT**	146	[127 199]	-0.30	[-0.37–0.23]	-0.68	[-0.90–0.58]	-1.87	[-2.05–1.62]
	**LIT**	**98**	**[56 135]**	**-0.29**	**[-0.34–0.25]**	**-0.66**	**[-0.79–0.58]**	**-1.84**	**[-2.00–1.71]**
	**FS**	367	[248 551]	-0.17	[-0.19–0.13]	-0.62	[-0.73–0.49]	-1.29	[-1.38–1.11]
	**SPM12**	312	[90 524]	-0.09	[-0.11–0.06]	-0.18	[-0.23–0.14]	-0.53	[-0.61–0.47]
	**KNBSI**	117	[95 149]	-0.21	[-0.23–0.18]	-0.70	[-0.76–0.57]	-1.53	[-1.59–1.42]
	**QUARC**	278	[98 529]	-0.17	[-0.22–0.12]	-0.61	[-0.71–0.47]	-1.29	[-1.44–1.11]
	**TBM**	216	[98 320]	-0.14	[-0.16–0.11]	-0.25	[-0.33–0.17]	-0.79	[-0.87–0.73]
**Lateral ventricles**	**CS**	173	[127 271]	1.39	[1.05 1.56]	4.46	[3.78 5.39]	10.03	[8.81 10.80]
	**IT**	214	[141 305]	1.18	[0.93 1.37]	3.90	[3.16 4.62]	8.64	[7.40 9.59]
	**LIT**	**148**	**[80 190]**	**1.30**	**[1.00 1.60]**	**3.86**	**[3.19 4.33]**	**9.04**	**[8.21 9.72]**
	**FS**	199	[108 287]	1.51	[1.12 1.84]	4.53	[3.68 5.64]	10.57	[9.66 11.51]
	**SPM12**	**145**	**[125 186]**	**0.89**	**[0.79 0.93]**	**2.36**	**[2.12 2.68]**	**5.93**	**[5.41 6.26]**
	**KNBSI**	199	[153 281]	1.50	[1.09 1.81]	4.46	[3.63 5.48]	10.47	[9.34 11.34]
	**QUARC**	167	[23 225]	1.84	[1.54 2.39]	4.67	[3.15 6.05]	12.02	[11.47 13.44]
**Right hippocampus**	**CS**	240	[123 353]	-0.58	[-0.68–0.49]	-1.48	[-1.74–1.18]	-3.81	[-4.19–3.39]
	**IT**	131	[14 205]	-0.57	[-0.74–0.46]	-1.09	[-1.28–0.83]	-3.38	[-3.87–3.01]
	**LIT**	**70**	**[52 90]**	**-0.65**	**[-0.73–0.57]**	**-0.82**	**[-1.05–0.55]**	**-3.43**	**[-3.62–3.18]**
	**FS**	191	[70 294]	-0.71	[-0.91–0.52]	-1.33	[-1.73–1.08]	-4.17	[-4.93–3.60]
	**SPM12**	>1000	[––]	-0.04	[-0.10 0.02]	-0.17	[-0.29 0.05]	-0.35	[-0.56–0.01]
	**KNBSI**	173	[67 280]	-1.26	[-1.62–0.87]	-0.68	[-6.43–4.90]	-5.73	[-0.25 0.54]
	**QUARC**	130	[93 166]	-0.59	[-0.65–0.51]	-0.98	[-1.16–0.76]	-3.32	[-3.55–3.01]
**Lefthippocampus**	**CS**	219	[190 317]	-0.62	[-0.68–0.47]	-1.44	[-1.93–1.16]	-3.94	[-4.27–3.41]
	**IT**	91	[66 130]	-0.60	[-0.69–0.45]	-1.10	[-1.35–1.01]	-3.48	[-3.83–3.10]
	**LIT**	**67**	**[43 88]**	**-0.61**	**[-0.70–0.52]**	**-0.93**	**[-1.18–0.77]**	**-3.38**	**[-3.65–3.20]**
	**FS**	140	[71 167]	-0.84	[-0.99–0.78]	-1.10	[-1.30–0.78]	-4.46	[-4.82–4.33]
	**SPM12**	>1000	[––]	0.02	[-0.05 0.06]	-0.16	[-0.37 0.10]	-0.08	[-0.30 0.05]
	**KNBSI**	194	[111 266]	-1.07	[-1.28–0.01]	-0.94	[-5.67–4.84]	-5.23	[-0.07 0.35]
	**QUARC**	133	[83 203]	-0.51	[-0.60–0.37]	-1.08	[-1.42–0.75]	-3.12	[-3.32–2.73]

**Fig 6:**
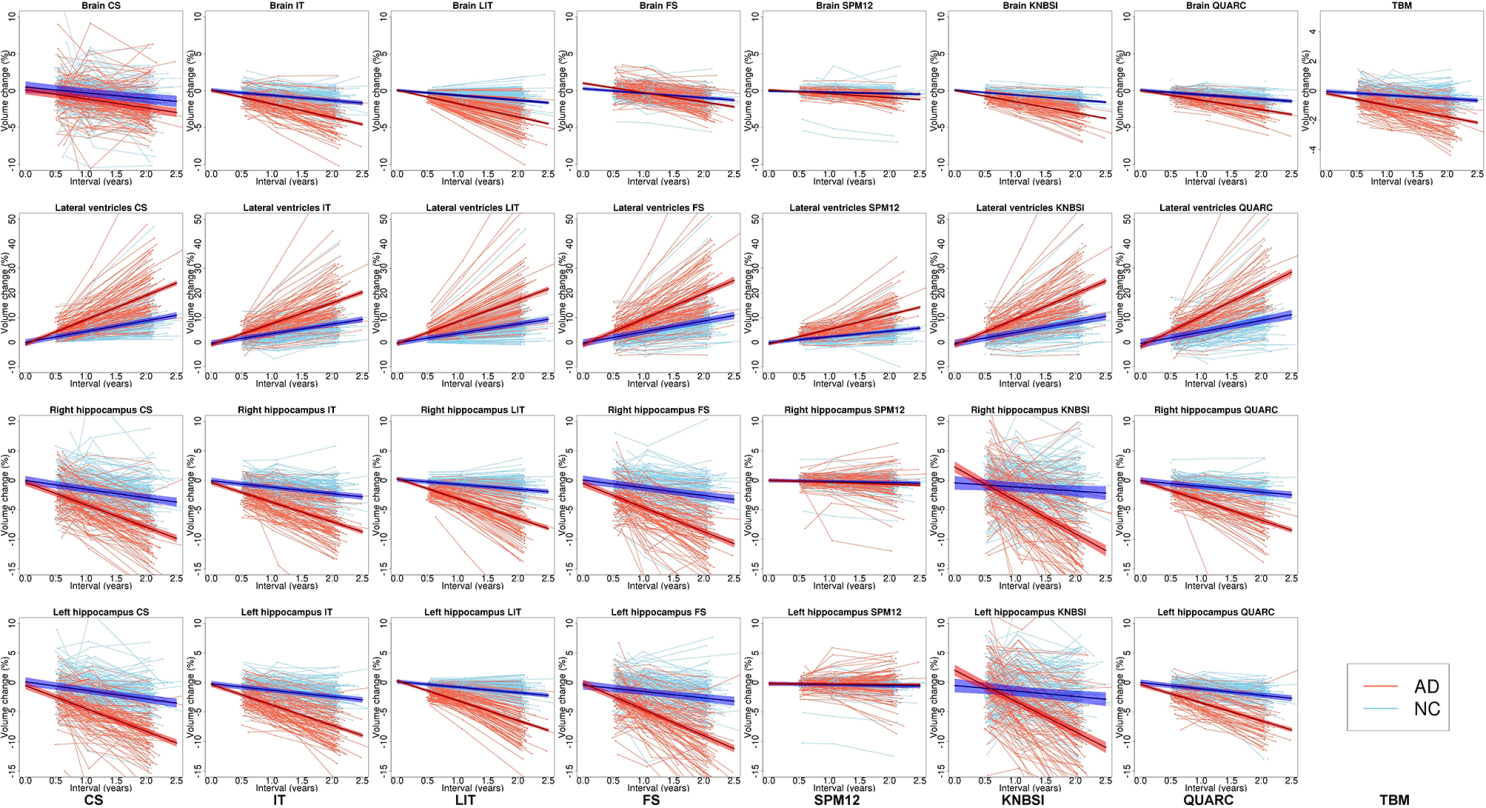
Longitudinal individual and linear mixed model with confidence intervals for the NC (blue) and AD (red) groups. Brain, ventricular and left/right hippocampi volume changes for CS, IT, LIT,FS, SPM12, KNBSI and TBM. Each thin full line represents an individual subject volume. Thicker lines represent the LME model for the respective groups while the shaded bands represent the 95% confidence interval on the mean model.

Fig 6 shows the individual longitudinal whole brain, ventricular and hippocampi changes (or cumulative atrophy) for each group (NC in blue and AD in red) and are described in more detail in the following sections.

#### Whole brain measurements

With regard to the whole brain, the LIT method results in a sample size of 98 to detect a 25% change in brain atrophy, versus 146 for IT and more than 1000 subjects required for the cross-sectional approach (CS). Furthermore, the LIT sample size is smaller than KNBSI (117) and TBM (216 subjects per arm). The LIT sample size range (56–135) overlaps with the following approaches: IT (127–199), KNBSI (95–149), QUARC (98–529) and TBM (98–320), however, LIT and IT provide a stronger effect size (-0.29 and -0.34) than these other methods.

Regarding the individual trajectories seen in the spaghetti plots in Fig 6, LIT provides a more progressive and regular individual trend while preserving group differences. It is interesting to note that the local constraints on the Jacobian over time result in a structure-wide regularization. KNBSI and QUARC measurements show a reduced individual longitudinal variability as well, compared to CS, FS, IT and TBM.

#### Lateral ventricle measurements

Among the different techniques tested, SPM12 and LIT yield the best power to detect a 25% reduction in lateral ventricular enlargement with only 145 and 148 subjects required per arm, but SPM12 shows the tightest range (125–186 and 80–190, respectively). The LIT effect size is stronger than SPM12 with a value of 1.30 versus 0.89 to discriminate the ventricular growth rate change between AD and NC. The CS approach of our pipeline yields better performance than the IT method (173 and 214 subjects, respectively), but the LIT reduces this number to 148 subjects.

When looking at the segmented lateral ventricle volumes in Fig 6, the trend of the observed ventricular enlargement is similar between the methods, but there is a net decrease of intra-subject variability for the longitudinal methods (IT, LIT, FS, SPM12, KNBSI and QUARC), as evidenced by spaghetti plots with more realistic, less chaotic changes over time. We can also appreciate with Fig 6. that the lateral ventricle volume changes are the strongest but also the more stable progression compared to other structures regardless of the method.

#### Hippocampus measurements

Among the different hippocampal methods tested, the LIT technique yields the best power to detect a 25% reduction in atrophy, with 67 subjects (left side) and 70 subjects (right side) required. When the temporal constraint is not applied to the deformations, the IT method requires 91 and 131 subjects (left and right side, respectively) to detect the same change. The other methods require more than 100 subjects to detect the same potential treatment effect. The estimation of the LME for SPM12 did not converge well enough to perform power analyses. FS shows the stronger effect size (-0.71±0.20 and -0.84±0.15 for the right and left hippocampi) but the effect size variability is much larger than for LIT (-0.65±0.08 and 0.61±0.09).

Fig 6 shows that the individual hippocampal trajectory variability is clearly decreased with the longitudinal methods, and in particular with IT, LIT and QUARC.

#### Jacobian maps

**Fig 7:**
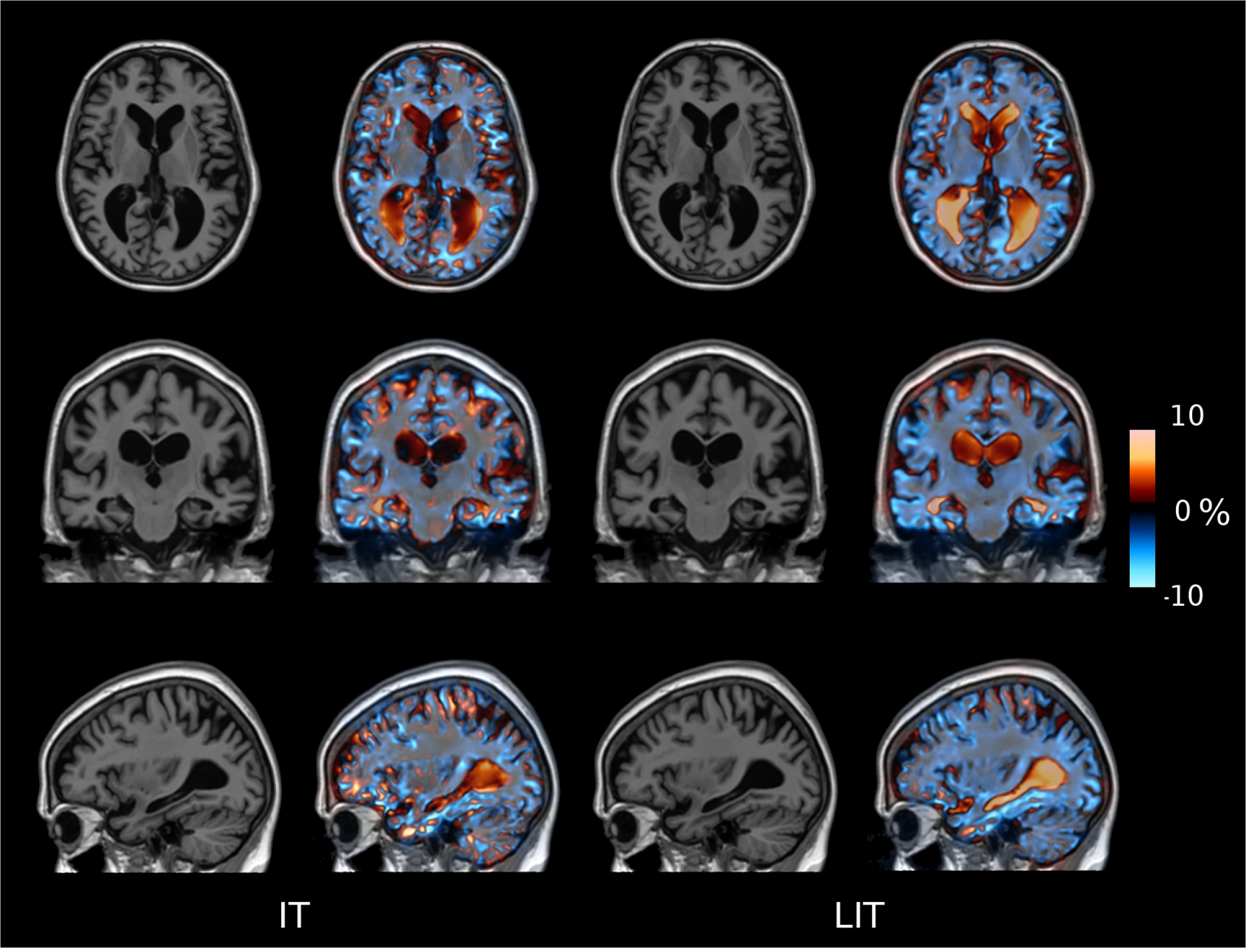
Longitudinal deformation fields. Deformation fields from baseline to the 12 month time-point for the longitudinal approaches (IT and LIT) where red represent growth and blue atrophy for a randomly chosen AD subject.

The concatenation of the transformation allows us to assess the total deformation between two specific time-points. Following this idea, Fig 7 shows the Jacobian of the determinant of the deformations estimated for the longitudinal methods (IT and LIT) for an AD patient. The IT Jacobian maps have multiple punctuate shrinking and enlarging regions within the ventricles that are not consistent with the notion of gradual ventricular growth that is relatively homogenous throughout the ventricle. By using a subject-specific template and the 4D regularization with the LIT methods (rightmost images), there are focal and consistent deformations that overlap well with the anatomy that is assumed to change with AD. Indeed, one can appreciate stronger temporal lobe atrophy detected with the LIT approach.

## Discussion

In this article, we have presented a new approach for the estimation of individual longitudinal changes using individual subject-specific templates and spatio-temporal regularization. We also provide an unbiased framework for analysing longitudinal data where every time-point is processed with the same steps. A robust estimation of the deformations is obtained using an individual template approach, minimizing deformations between subject time-points. Meanwhile, a local spatio-temporal regularization is achieved with linear regression of the deformation field and a spatial regularization of its Jacobian matrices. The regression of the decomposition enables a temporal regularization at a local voxel level. Furthermore, we compared our technique with a traditional cross-sectional approach, as well as recent powerful methods, FS, SPM12, KNBSI, QUARC and TBM. Longitudinal image analysis bias was assessed on a scan-rescan dataset, and power analysis to detect a potential treatment effect on an Alzheimer cohort was chosen.

Longitudinal image analyses can be subject to bias in particular due to non-linear registration when an arbitrary reference image is chosen [6] or due to interpolation asymmetry [57]. 4D Hammer [58] or non-linear registration such as Diffeomorphic Demons [13, 59] and ANTs [60] require a reference image to be defined, therefore introducing possible bias. Symmetric interpolation and registration might not be sufficient to correct for bias when there are more than 2 time-points. The use of an individual template, as suggested by Reuter et al. [16] showed no bias and our approach exploits this strength and adds non-linear registration to obtain a more accurate anatomical correspondence between time-points.Then, the individual template can be used to segment brain structures directly and not only to initialize the segmentation as it is done in FS. Here, we divided the estimation of a linear and a non-linear individual template, but other approach for group-wise registration have proposed to estimate more complex deformation models such as free-from deformations where both linear and non-linear deformations are combined into a single model [61].

By definition, our approach, using a longitudinal pre-processing to remove interpolation bias and an individual template for non-linear registration, is symmetric and transitive as it is similar to a “half-way” space registration approach [62]. Indeed, the longitudinal pre-processing applies the same number of interpolations and removes the intensity inter-visit non-uniformity. Furthermore, the non-linear registration is performed towards a common target, producing unbiased deformation fields that can be combined to obtain robust and transitive non-linear deformations between time-points. The individual template estimation depends of the current set of the subject’s time-points, thus the addition of new time-points will require a re-estimation of the individual template. This could limit its use in clinical settings where intermediate results are expected before the MRI acquisition of the final time-point.Future work should focus on the longitudinal measurement stability when an additional time-point is pre-processed and registered to the specific template previously estimated separately.

In our experiments, we have demonstrated that the LIT method provides a more robust longitudinal measure on a scan-rescan dataset where no changes are expected. We have also found that by using individual subject-specific templates (IT, LIT, FS and SPM12), structure volume variability is decreased compared to the cross-sectional approaches like CS that use a single common template (e.g., the ICBM152 model) for all subjects. Among the three longitudinal methods tested, the LIT and SPM12 demonstrated the least bias and the smallest variability in structure volumes which is expected since both methods apply a longitudinal regularization and therefore minimize the temporal variability.

Experiments on ADNI data reveal increased stability in estimating individual changes over time compared to standard cross-sectional approaches. Indeed, the cross-sectional approach was chosen as a reference method and allowed us to show an important improvement in the measurement of longitudinal change thanks to the longitudinal pre-processing (IT) and temporal regularization constraint (LIT). However, other strictly cross-sectional approaches with independent time-point measures have showed to perform better in a similar study such as FS in Holland et al. [47]. Furthermore, the longitudinal approaches exploiting the full study length (4 time-points) allowed to improve the power when compared to pair-wise (2 time-points) approaches suggesting that the improvement could be related to the additional time-points themselves.

The longitudinal regularization of the deformation *at a local level* reduces the longitudinal noise in volume estimation *at the global/structural level*, while the hierarchical iterative process produces a robust individual template that allows for better anatomical matching across time in an individual. An important aspect of longitudinal clinical and research studies is the cost of recruiting subjects and scanning them at multiple time-points. The proposed longitudinal analysis techniques will allow for better power to detect differences between groups, and thus will lead to the reduction of the number of subjects required for research and for clinical trials. Compared to the literature, where similar ADNI cohorts of AD and NC were used, our power analysis shows similar sample sizes required to detect treatment effects for the FS, KNBSI, QUARC and TBM approaches [47]. The proposed temporal constraint (LIT), reduces the sample sized by a factor by approximately 50% for brain, 70% for the lateral ventricles and 50% for hippocampi when compared to the similar longitudinal pre-processing without spatio-temporal regularization (the ID approach). The temporal constraint from SPM12, developed to optimize longitudinal VBM, produces unbiased results on the scan-rescan dataset but might be over regularizing the longitudinal deformation to detect structural change on the ADNI cohort in this experiment. Indeed, it is important to mention that smoothing the temporal fluctuation could remove temporal artefacts, while it could also smooth real signal fluctuations. Within LIT, the linear longitudinal constraint is only applied at a voxel level, i.e., the displacement of a point in the brain is constrained to move in a linear fashion over time. But the volume of the structure is not explicitly constrained to continuously increase or decrease. This constraint results in globally more continuous volume changes, which are not the result of an explicit constraint on the volume. Indeed, the increase in statistical power to detect group difference using LIT suggests a reduction of the longitudinal variability but not at the expense of small changes detection.

With more time-points and longer studies, the regularization could be adapted to fit other models to capture the potential longitudinal changes (ie. exponential or polynomial regressor).Similarly, in the context of patient classification using Support Vector Machine (SVM), spatial and anatomical regularization techniques (Sobolev, LASSO…) have shown to improve the classifier accuracies in the presence of noise [63, 64].

Another interesting finding is that longitudinal pre-processing and individual template creation does not affect the longitudinal measurements of anatomical structures in the same manner. Indeed, structures such as the lateral ventricles, with high contrast and less sensitive to bias field and distortion, resulted in a similar sample size for both longitudinal and cross-sectional approaches. However, the spatio-temporal regularization is able to decrease the longitudinal variability of such structures and therefore reduce the sample size.

We limited our comparison to publicly available methods and/or results on the ADNI-1 cohort, but other methods have been developed and applied on real longitudinal data. The complexity and/or the computational cost of these methods [10, 14, 65] may limit the application to large databases such as ADNI. Wu et al. [14] aligned all longitudinal images of a population toward a hidden common space equivalent to a template and it can be applied to a single subject. The individual longitudinal deformations or “temporal fibers” are estimated without any priors but regularized with a Gaussian kernel to preserve the continuity of the longitudinal deformation field. Similarly, Lorenzi et al. [13] proposed to fit a linear model to constrain the longitudinal velocity fields of the subjects time-points in the Demons’ framework with the baseline image used as a reference. Despite the fact that their approaches aremore general in modelling the deformations, the usual small number of time-points might limit the longitudinal continuity.

Finally, the main focus of this article was to compare longitudinal regularization versus longitudinal pre-processing and cross-sectional approaches. Although we focused on whole brain, lateral ventricles and hippocampi, any other structures can be analyzed longitudinally as far as the individual template can be segmented. The longitudinal Jacobian determinant maps show interesting results to measure voxel-wise deformation individually with the spatio-temporal regularization. The deformation maps present plausible anatomical atrophies such as in gray matter and in temporal lobes as well as a uniform ventricular enlargement. The results are encouraging and hold the potential of voxel-wise longitudinal DBM of neurodegenerative diseases.

Finally, the proposed longitudinal pipeline will be made available online (https://www.mcgill.ca/bic/software/tools-data-analysis/anatomical-mri/) and relies on the publicly available MINC tools and library (https://www.mcgill.ca/bic/software/minc).

## Conclusion

This study evaluated a longitudinal framework with spatio-temporal regularization of deformation fields and the creation of an individual 3D template through non-linear registration in the context of longitudinal neuroimaging studies. The experiments were carried out on scan-rescan and ADNI datasets. In comparison with freely available and popular methods, the spatio-temporal regularization (LIT) shows competitive results in regard to robustness, power and stability while reducing the number of subjects required to show statistical differences between groups. In addition, the LIT approach showed promising results for longitudinal DBM analysis and can be easily adapted to investigate specific anatomical biomarkers.
